# Lung Mast Cells Have a High Constitutive Expression of Carboxypeptidase A3 mRNA That Is Independent from Granule-Stored CPA3

**DOI:** 10.3390/cells10020309

**Published:** 2021-02-03

**Authors:** Premkumar Siddhuraj, Carl-Magnus Clausson, Caroline Sanden, Manar Alyamani, Mohammad Kadivar, Jan Marsal, Joanna Wallengren, Leif Bjermer, Jonas S. Erjefält

**Affiliations:** 1 Department of Experimental Medical Sciences, Lund University, 221 84 Lund, Sweden; premkumar.siddhuraj@med.lu.se (P.S.); carl_magnus.clausson@med.lu.se (C.-M.C.); caroline.sanden@med.lu.se.se (C.S.); manar.alyamani@med.lu.se (M.A.); mokad@dtu.dk (M.K.); 2 Medetect AB, Medicon Village, 223 81 Lund, Sweden; 3 Department of Gastroenterology, Lund University, Skane University Hospital, 221 85 Lund, Sweden; jan.marsal@med.lu.se; 4 Department of Dermatology, Lund University Skane University Hospital, 221 85 Lund, Sweden; joanna.wallengren@med.lu.se; 5 Department of Allergology and Respiratory Medicine, Lund University, Skane University Hospital, 221 85 Lund, Sweden; leif.bjermer@med.lu.se

**Keywords:** mast cells, carboxypeptidase A3, lung, gut, skin, chymase, tryptase, mRNA

## Abstract

The mast cell granule metalloprotease CPA3 is proposed to have important tissue homeostatic functions. However, the basal CPA3 mRNA and protein expression among mast cell populations has remained poorly investigated. Using a novel histology-based methodology that yields quantitative data on mRNA and protein expression at a single-cell level, the present study maps CPA3 mRNA and protein throughout the MCT and MCTC populations in healthy skin, gut and lung tissues. MCTC cells had both a higher frequency of CPA3 protein-containing cells and a higher protein-staining intensity than the MCT population. Among the tissues, skin MCs had highest CPA3 protein intensity. The expression pattern at the mRNA level was reversed. Lung mast cells had the highest mean CPA3 mRNA staining. Intriguingly, the large alveolar MCT population, that lack CPA3 protein, had uniquely high CPA3 mRNA intensity. A broader multi-tissue RNA analysis confirmed the uniquely high CPA3 mRNA quantities in the lung and corroborated the dissociation between chymase and CPA3 at the mRNA level. Taken together, our novel data suggest a hitherto underestimated contribution of mucosal-like MCT to baseline CPA3 mRNA production. The functional consequence of this high constitutive expression now reveals an important area for further research.

## 1. Introduction

The mast cell is a key immune cell that, apart from having key roles in both innate and adaptive immunity, also possesses important roles in tissue homeostasis [1,2,3]. Mast cells are laden with characteristic granules that serve as storage sites for a variety of ready-made effector molecules [4]. Among these are potent proteases, some of which are also used to define mast cell subpopulations. Thus, based on the tryptase and chymase profile, mast cells are classically divided into “connective tissue like” mast cells (MCTC cells (containing tryptase and chymase)) and “mucosal like” mast cells (MC_T_, lacking chymase) [1,2,3].

The focus on tryptase and chymase for MC classification also reflects the many important roles assigned to these proteases [4,5,6,7]. However, mast cell granules contain other less explored proteases, which are likely to have significant biological roles, such as cathepsin G, renin, MMP9, active caspase 3, and carboxypeptidase A3 (CPA3) [4]. Interestingly, CPA3 has recently been highlighted in multiple gene signature studies where CPA3 expression has been linked to eosinophilic esophagitis [8,9], colon cancer [10], and eosinophilic type 2 asthma [11,12,13]. Although these observations indicate clinically important roles for CPA3, there is limited information regarding its substrate cleavage profile. CPA3 is a zinc metalloprotease and, to date, known and tentative targets for cleavage include neurotensin, kinetensin, endothelin-1, apolipoprotein B, and angiotensin I [14,15,16,17,18,19]. From this target list, it seems that CPA3 is not only involved in pathological processes but also has roles in the homeostatic regulation of, e.g., the vasculature and matrix turnover.

Generally, CPA3 protein storage is thought to be linked to MCTC cells [4,20]. This relationship may be explained by observations of shared mechanisms for granule packaging between chymase and CPA3 [21,22]. However, this interdependence and co-localization seems not to be strict, since subsets of MCs containing CPA3 but lacking chymase have been observed in asthmatic airways [23], allergic rhinitis [24] and eosinophilic esophagitis [25,26]. These observations indicate that CPA3 expression may be more complex than previously thought. Addressing this issue, given that CPA3 expression seems to be influenced by the anatomical and microenvironmental context, a prudent approach may be to explore the baseline expression heterogeneity of CPA3 at both the mRNA and protein levels in normal human tissues. In fact, surprisingly little is known about the basic dynamics and interplay between CPA3 mRNA and protein expression in tissue mast cells. The present study represents the first such detailed exploration and is based on a histological methodology that yields quantitative data on mRNA and protein expression at a single-cell level with full tissue-spatial resolution. Using this approach, we reveal critical new information on the dynamics and interplay between CPA3 mRNA expression and the granule-stored CPA3 protein level, across MCT and MCTC mast cells in normal human tissues, including lung, colon, and skin.

## 2. Materials and Methods

### 2.1. Human Tissue Acquisition and Sample Processing

In order to avoid any potential technical bias inflicted by the variation in, e.g., fixation times, that commonly occurs in routine clinical pathology labs, all excised tissue samples in this study were prepared at our own research laboratory and immediately subjected to controlled and standardized fixation in buffered 4% formaldehyde (#02176; Histolab, Askim, Sweden) for 24 ± 4 h at room temperature before being dehydrated in an automated dehydration machine (SAKURA-Tissue-Tek VIP^®^ 6 AI; Sakura Finetek USA, Inc., Torrance, CA, USA) and embedded into paraffin blocks (SAKURA EC350; Sakura Finetek, Inc, Torrance, CA, USA). From each patient, several cm^2^ -sized 4 µm paraffin sections were generated, dehydrated and used for the present high-end quantitative protease analysis. The following tissues were collected:

*Human lung tissue*: Non-diseased human control lung tissue was obtained in from 10 non-atopic patients undergoing lobectomy due to suspected lung cancer but otherwise lacking any history of respiratory disease. Only patients with solid, well-delineated tumors were included and central and distal lung tissue samples were collected as far as possible from the tumor site. Although the majority of the subjects are former smokers, this procedure has commonly been used to collect non-diseased control lung tissue samples for histological research [27,28]. All patients (*n* = 10) gave their written informed consent, which was approved by the ethical committee in Lund, Sweden (Dnr. 2018/54);

Human Large Intenstine: Tissue biopsies were collected from patients investigated with endoscopy due to gastrointestinal symptoms. For this study, biopsies from patients (*n* = 7) with normal findings on the colonoscopy, and where any inflammatory disorder was discarded by the clinical pathological examination and follow-up clinical data, were included. All patients gave their written informed consent, which was approved by the ethical committees in Lund and Linköping, Sweden (Dnr. 2011/60 and 2011-201-31, respectively);

*Human Skin:* Using a routine punch biopsy tool, skin biopsies were collected from healthy-looking skin from control patients (*n* = 6). All patients gave their written informed consent, which was approved by the ethical committee in Lund, Sweden (Dnr. 2011/151).

### 2.2. Triple Immunofluorescence for Quantitative Assessment of CPA3 in MCT and MCTCs

As part of the antigen retrieval process, slides were baked at 60 °C for 45 min and pretreated with low pH heat-induced epitope retrieval (HIER) by a pH6 target retrieval solution (#DM829; Dako, Glostrup, Denmark) in a DAKO PT Link HIER machine (PT-link 200; Dako, Glostrup, Denmark). Immunofluorescence triple staining was achieved using an automated immunohistochemistry robot (Dako Cytomation, Glostrup, Denmark). Pretreated slides were rinsed thoroughly in wash buffer (#DM831; Dako, Glostrup, Denmark) and blocked with the serum-free solution (#X0909; Dako, Glostrup, Denmark) for 10 min. For the bulk quantification of tryptase, chymase and CPA3, the following triple immunofluorescence protocol was used: After treatment with a biotin blocking solution (#X0590, Dako, Glostrup, Denmark) for 20 min, slides were rinsed with wash buffer, incubated with a rabbit anti-CPA3 primary antibody (#HPA008689, 0.1 mg/mL; dilution 1:1000; Atlas Antibodies, Bromma, Sweden), rinsed, and then incubated for 60 min with a biotin-conjugated Goat anti-Rabbit secondary antibody (#BA-1000, dilution 1:200; Vector Laboratories, Inc.; Burlingame, CA, USA), followed by a rinsing step and labelling of the biotin with AlexaFlour 647-streptavidin (#S21374, 1 mg/mL; Thermo Fisher Scientific, Waltham, MA, USA) for 30 min. Next, any potential capacity of the rabbit primary antibody that was “accidentally” recognized by subsequent secondary anti-mouse antibodies was incapacitated by a 5 min denaturating blocking step (#DNS 001; Biocare Medical, Pachecho, CA, USA), followed by a rinsing step. Thereafter, sections were incubated with a cocktail of mouse anti-human tryptase (#MAB1222A; 0.1 mg/0.1 mL, dilution 1:350; Millipore, Burlington, MA, USA) and a rabbit anti-human chymase primary antibody (#HPA0526634; 0.4 µg/mL, dilution 1:500; Atlas Antibodies, Bromma, Sweden). After a washing step, these primary antibodies were detected by a secondary antibody cocktail with an Alexa Fluor 555 Goat anti-Rabbit antibody (#A21428; 2 mg/mL, 1:200; Thermo Fisher Scientific, Göteborg, Sweden) and an Alexa Fluor 488 Donkey anti-Mouse secondary antibody (#A21206; 2 mg/mL, 1:200; Thermo Fisher Scientific, Göteborg, Sweden).

### 2.3. Staining mRNA Separately and in Combination with Proteins in MCT and MCTC Populations for Subsequent Computerized Quantification

Visualization of CPA3 mRNA was performed through in situ hybridization (ISH) using the RNAscope^®^ Multiplex fluorescence kit V2 assay kit (#323100; Advanced Cell Diagnostics, Hayward, CA, USA) for fluorescent ISH (FISH) and using the RNAscope^®^ 2.5 HD Detection Reagents-RED (#322360, Advanced Cell Diagnostics) for chromogenic ISH (CISH), according to the manufacturer’s instructions. Briefly, 4 µm lung tissue sections were baked at 60 °C for 1 h and then deparaffinized through a series of alcohol and xylene baths. Afterwards, the slides were incubated with hydrogen peroxide for 10 min at RT and then with an mRNA retrieval solution for 15 min at 99 °C before dehydration with 99.5% EtOH for 3min at RT, and then evaporation at 60 °C for 5 min. After creating a hydrophobic border around each tissue specimen, they were incubated at 40 °C for 30 min, together with RNAscope Protease Plus, and then at the same temperature for 2 h with the CPA3 mRNA probe (#486731). Additional mRNA probes were run in parallel: Tryptase (TPSAB1, #313901; note that although this probe is designed against TPSAB1, it may also recognize the closely related TPSB2 (beta II tryptase) mRNA sequence), Chymase (CMA1; #486721) negative control (DapB, #310043;), positive control probe (PPIB, #486081; Advanced Cell Diagnostics). All mRNA probes were purchased from Advanced Cell Diagnostics. Amplification and detection probes were then stepwise incubated with the samples according to instructions. For fluorescent read-out, TSA amplification was performed with Cyanine 3 (NEL744E001KT, Perkin Elmer, Waltham, MA, USA). For chromogenic read-out, the red chromogen included in the aforementioned RNAscope^®^ 2.5 HD kit was used. The slides with fluorescent read-out were then blocked with an HRP blocker and placed in PBS awaiting immunofluorescence staining. The slides with chromogenic read-out were counterstained with Mayer’s hematoxylin (#01820; Histolab, Askim, Sweden) for 45 s, before rinsing in dH2O and incubation in saturated LiCO3 for 5 s, another rinsing step in dH2O and subsequent evaporation at 60 °C for 20 min before mounting with pertex (#00840; Histolab, Askim, Sweden) and No.1 coverslips (ECN 631-1574; VWR, Radnor, PA, USA). After the fluorescence in situ hybridization of CPA3 mRNA in the cy3 channel, slides were subjected to subsequent immunofluorescence staining for tryptase and chymase proteins in cy2 and cy5 channels, respectively. For tryptase and chymase immunostainings, sections were incubated with a cocktail of a mouse anti-human tryptase (#MAB1222A; 0.1 g/0.1 mL, dilution 1:350; Millipore, Solna, Sweden) and a rabbit anti-human chymase primary antibody (#HPA0526634; 0.4 µg/mL, dilution 1:500; Atlas Antibodies, Bromma, Sweden). After a washing step, these primary antibodies were detected by a secondary antibody cocktail with an AlexaFluor 647 Goat anti-Rabbit secondary antibody (#A27040; 2 mg/mL, 1:200; Thermo Fisher Scientific, Göteborg, Sweden) and an Alexa Fluor 488 Donkey anti-Mouse secondary antibody (#A21206; 2 mg/mL, 1:200; Thermo Fisher Scientific, Göteborg, Sweden).

All the primary and secondary antibodies were diluted in antibody diluent (#S0809, Dako). After completing IF staining, the slides were incubated with Bisbenzimide-33342 (#A0741; Applichem, Darmstadt, Germany) for nucleus counterstaining and mounted with Vectashield (#H1400; Vector Laboratories, Inc, Burlingame, CA, USA) and No.1 coverslips (ECN 631-1574; VWR, Radnor, PA, USA). The present antibody against CPA3, and the other primary and secondary antibodies, have been validated and used in multiple previous studies [23,24]. Careful tests also confirmed the specificity of the staining results, i.e., that there were no artifactual cross-reactions and unwanted streptavidin binding, and that the low autofluorescence within mast cells had no or negligible impact on the analyses.

### 2.4. Slide Digitalization and IF Image Quantification

The Olympus VS-120 (Shinjuku, Tokyo, Japan) fluorescence virtual microscopy slide-scanning platform was used for digitalization of fluorescence-stained tissue sections. The fluorophores corresponding to DAPI, FITC, CY3 and CY5 channels were recorded with a “locked” exposure time for each channel across all the samples within an experiment. Separate test experiments confirmed minimal bleeding between the fluorescence filter settings. Digitalized images were converted from its original format (Olyvia-VSI files) to 16-bit Big-TIFF GB-size files for subsequent image processing and quantification. In order to measure CPA3 protein and mRNA content at the single-cell level, a mask was created for MCT and MCTCs. First, the mask for MCTC regions of interest (ROI) was segmented by applying a standard intensity threshold for the chymase channel. Secondly, the masks for MCTC from the chymase channel were superimposed on the tryptase channel and cells positive for both chymase and tryptase were eliminated, thus leading to true MCT ROIs.

Next, the resulting MCT and MCTC masks were virtually superimposed on to the raw data image for each original fluorescence channel to measure the mean fluorescence intensity for the corresponding CPA3 mRNA and protein stain within each individual ROI at a 0–16,000 dynamic range. Finally, after ROI size filtration, the intensity values were corrected by subtracting the tissue background fluorescence value.

To compare protease profiles within defined anatomical compartments, the following structures were manually delineated by cursor tracing: large and small airway compartments (i.e., bronchial and bronchiolar); epithelium and subepithelial tissue; large pulmonary vessels; alveolar parenchyma region. The ROIs for all compartments were applied to other channels of the same image to extract the single-cell data, as described above. For spatial visualization of mast cell distribution in lung tissue sections, X and Y coordinates were extracted during the quantification and superimposed on the original image. All of the above image analysis steps were performed using ImageJ software.

### 2.5. Data Visualization and Statistics

All results are presented as median with interquartile range and ranks were compared among samples using the non-parametric Mann–Whitney test. Symbols used for indicating statistical significance include: ns, not significant, *p* > 0.05, * *p* ≤ 0.05, ** *p* ≤ 0.01, and *** *p* ≤ 0.001. Calculations and graphs were performed using Prism version 6.01 (GraphPad, San Diego, CA, USA). JMP pro 15 statistical software was used to plot shadowgrams with normal distribution fit line.

## 3. Results

### 3.1. MCTC Cells Have Both a Higher Proportion of CPA3 Containing Cells and Significantly Higher Staining Intensity than Cells within the MCT Populations

**Figure 1:**
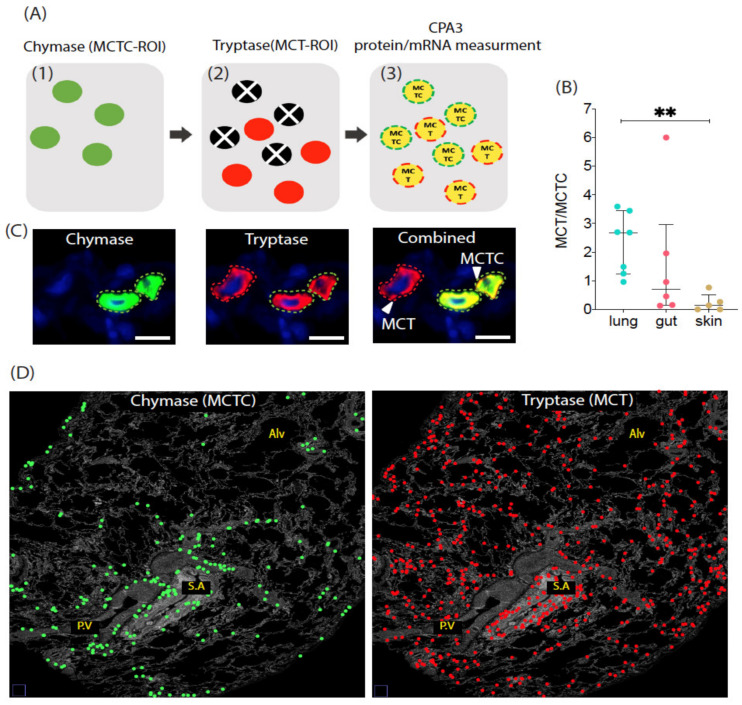
(**A**) A schematic overview of the principle used to quantify the human lung-, gut- and skin-tissue-residing MCT (tryptase^+^, chymase^-^) and MCTC (tryptase^+^, chymase^+^) mast cell subpopulations. The mRNA and protein were measured at the single-cell level by performing fluorescence in situ hybridization (FISH) and subsequent immunofluorescence staining. Following the staining procedure, slides were digitalized into high-resolution 16-bit images. Next, combined chymase and tryptase Regions of Interest (ROIs) with tryptase and/or chymase were segmented by a locked-intensity threshold. Finally, the combined MC ROIs were categorized as MCT (tryptase only) and MCTC (tryptase + chymase) objects, which were used as guiding points to quantify the mRNA and protein of interest per cell object basis using a semi-automated ImageJ platform. (**B**) The proportion of MCT and MCTC in lung, gut and skin. Statistical difference levels were calculated by Statistics–Dunn’s multiple comparisons test ns-*p* > 0.05, ** *p* ≤ 0.01, (**C**) Immunofluorescence micrographs of close-up single and combined tryptase and chymase cell profiles. (**D**) Micrographs exemplifying part of a lung surgical resection section area where the spatial distribution of 229 segmented MCTC and 637 MCT masks (ROIs) is illustrated by color-coded circles. Alv, alveolar parenchyma; PV, pulmonary vessels; SA, small airways (i.e., bronchioles).

Our principle for large-scale quantification of the protease content in individual mast cells in 2D histological sections is based on the use of mast cell-specific single-cell ROIs as guiding points for measuring the fluorescence intensity of CPA3 immunoreactivity for all hundreds to thousands of individual MCT and MCTC cells present per section (Figure 1A,C–D). In short, CPA3 staining intensity was measured in the cell marker objects (ROIs) containing chymase and tryptase (i.e., MCTC cells) or only tryptase (MCT cells) and is expressed as mean fluorescence intensity for the CPA3-specific wavelength channel. Lung, gut and skin were selected in order to include tissues with various baseline proportions of MCT and MCTC cells. As expected from the previous literature, MCT cells dominated in the lung, showed similar numbers as MCTC cells in the gut, and represented a small minority in the skin (Figure 1B).

**Figure 2:**
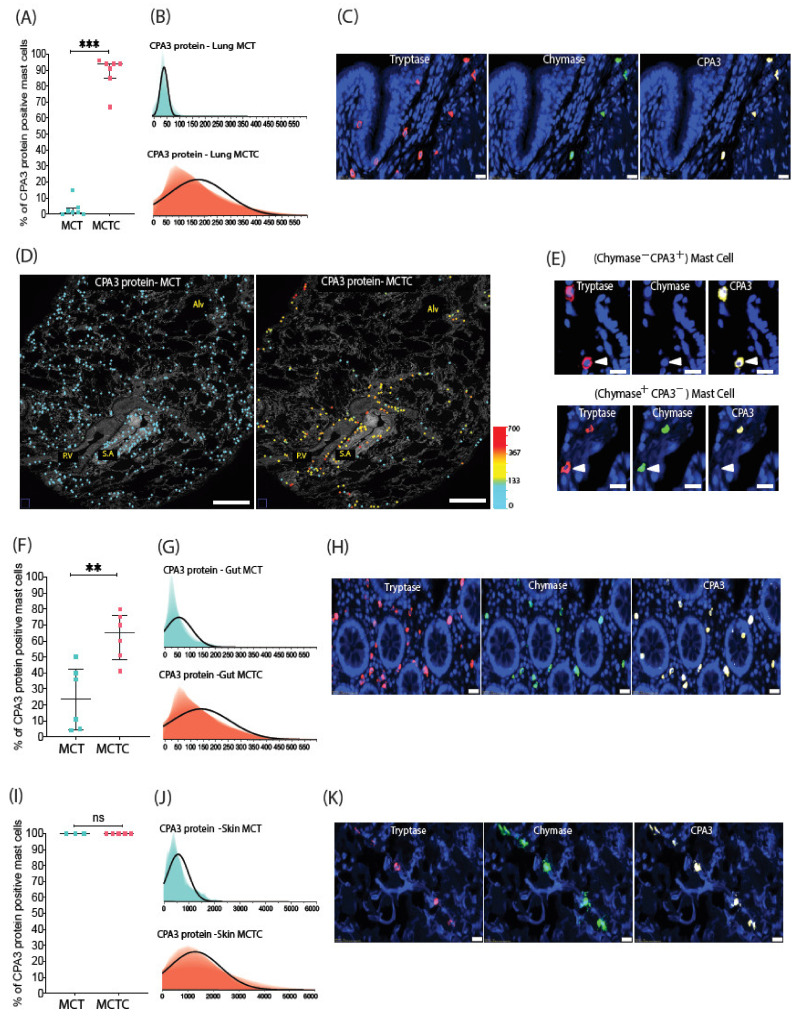
Mapping of CPA3 protein in MCT and MCTC populations. (**A**) Percentage of CPA3-protein-positive MCT and MCTC in lungs. Circles denote mean subject values (**B**) Shadowgrams (overlayed histograms with various X axis bin widths) exemplifying the cell frequency distribution across CPA3 protein intensity levels among 10 126 pooled individual MCT and MCTC cells (**C**) Immunofluorescence images showing the typical CPA3-positivity among lung MCTCs. (**D**) Low magnification micrographs displaying the spatial distribution of MCT and MCTC cells where cell x, y coordinates are marked with circles pseudocolored according to CPA3 intensity levels. (**E**) Immunofluroscence images exemplifying the rare CPA3^+^ MCT cells. (**F**,**G**) Mean subject proportion of gut MCT and MCTC cells positive for CPA3 protein, and shadowgrams of 1015 pooled gut MCs. Although MCTC display a significantly higher CPA3 protein level, a moderate amount of MCT test positive for CPA3 protein (whereas in lungs, MCTs are rarely positive for CPA3 protein). (**I**–**J**) The proportion of skin MCTs and MCTCs positive for CPA3 protein (a total of 705 cells were analyzed). Contrary to lung and gut mast cells, both MCT and MCTC skin mast cell subpopulations display an equal level of CPA3 protein. Immunofluorescence images exemplifying protease profiles for (**H**) gut and (**K**) skin mast cells expressing CPA3 protein. *Statistics:* (**A**,**F**,**I**) Mann–Whitney test used to calculate the significance of among mast cell subpopulation in relation to CPA3 protein expression, ns (non significant) -*p* > 0.05, ** *p* ≤ 0.01, *** *p* ≤ 0.001. (**B**,**G**,**J**) Shadowgrams were fitted using normal distribution line. Alv, alveolar parenchyma; PV, pulmonary vessels; SA, small airways (i.e., bronchioles).

The detailed single-cell CPA3 quantification revealed that in the lung, CPA3 protein was present predominantly within the MCTC population (Figure 2). Although few, some scattered CPA3-positive MCT cells were observed in the lung tissue (exemplified in Figure 2E). However, these MCT cells had a CPA3 low-expressing phenotype. From color-coded spatial overview images, it was clear that the CPA3 proteins containing MCTCs were mainly localized to the bronchiolar wall, larger pulmonary vessels and the visceral pleura (Figure 2D; note also the widespread distribution of CPA3-negative MCT cells in the alveolar parenchyma). A similar pattern as in the lung was observed in the gut, but the CPA3-expressing MCTs were more abundant and displayed higher mean CPA3 intensity levels (Figure 2F–G). Contrasting the lung and gut, skin mast cells had a very high CPA3-staining intensity, a feature also seen among the few MCTs present in skin (Figure 2I–J).

### 3.2. Contrasting the Predominance of CPA3 Protein in MCTCs, CPA3 mRNA Is Similar or Even Higher in the MCT Population

**Figure 3:**
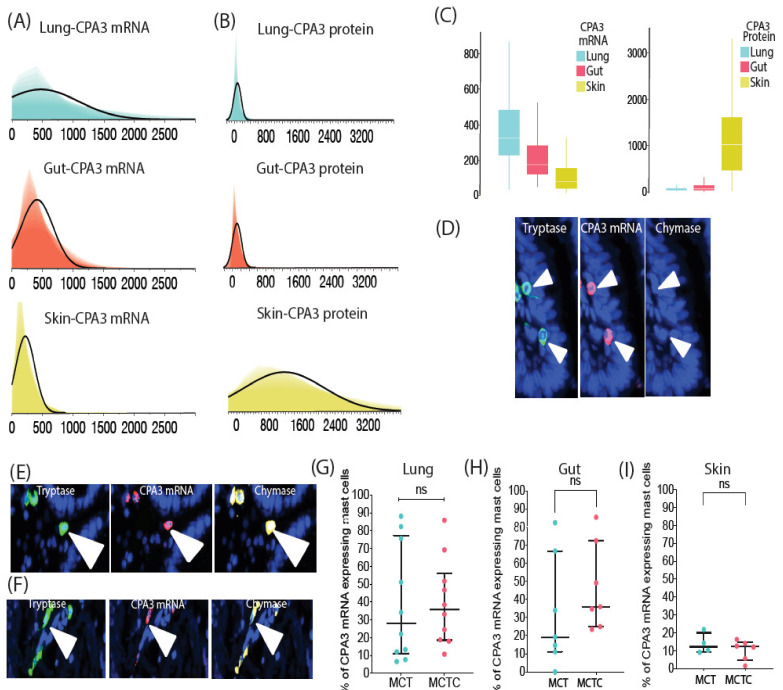
CPA3 mRNA expression in lung, gut and skin MCT and MCTC populations and inverse correlation of CPA3 mRNA with its protein storage in the granules. (**A**,**B**) Shadowgrams demonstrating the reversed expression patterns of staining intensity levels (y-axis) between CPA3 mRNA (**A**) and protein (**B**) in lung, gut and skin mast cells. Lung and gut contain significantly higher levels of CPA3 mRNA than skin (**B**). (**C**) The candlestick bar graphs illustrate further the reversed pattern of CPA3 mRNA and protein level between lung, gut and skin mast cells. (**D**–**F**) Immunofluorescence micrographs exemplifying CPA3 mRNA expression in lung, gut and skin mast cells, respectively. (**G**–**I**) Subject mean percentage of CPA3 mRNA expression by lung, gut and skin MCT and MCTC populations. *Statistics*: (**G**–**I**) Mann–Whitney test used to calculate the significance of among mast cell subpopulation in relation to CPA3 protein expression, ns = non-significant difference, i.e., *p* > 0.05.

Next, we applied a combined ISH-IF approach to study the relationship between CPA3 mRNA and protein between the examined tissues. The results unveiled a surprising but clearly reversed pattern where the extensive protein content in the skin was linked to low underlying mRNA production. The low level of stored CPA3 protein in the lung was associated with a particularly high and robust mRNA expression, while gut tissue displayed an intermediate pattern (Figure 3A–C). We also explored to what extent the level of CPA3 mRNA expression reflects the almost exclusive presence of granule-stored CPA3 protein in the MCTC population. Intriguingly, contrasting the protein pattern, CPA3 mRNA intensity levels were for all tissues at least as high in MCT cells as in MCTC cells and no statistical difference was found between the MC populations (Figure 3D–I).

**Figure 4:**
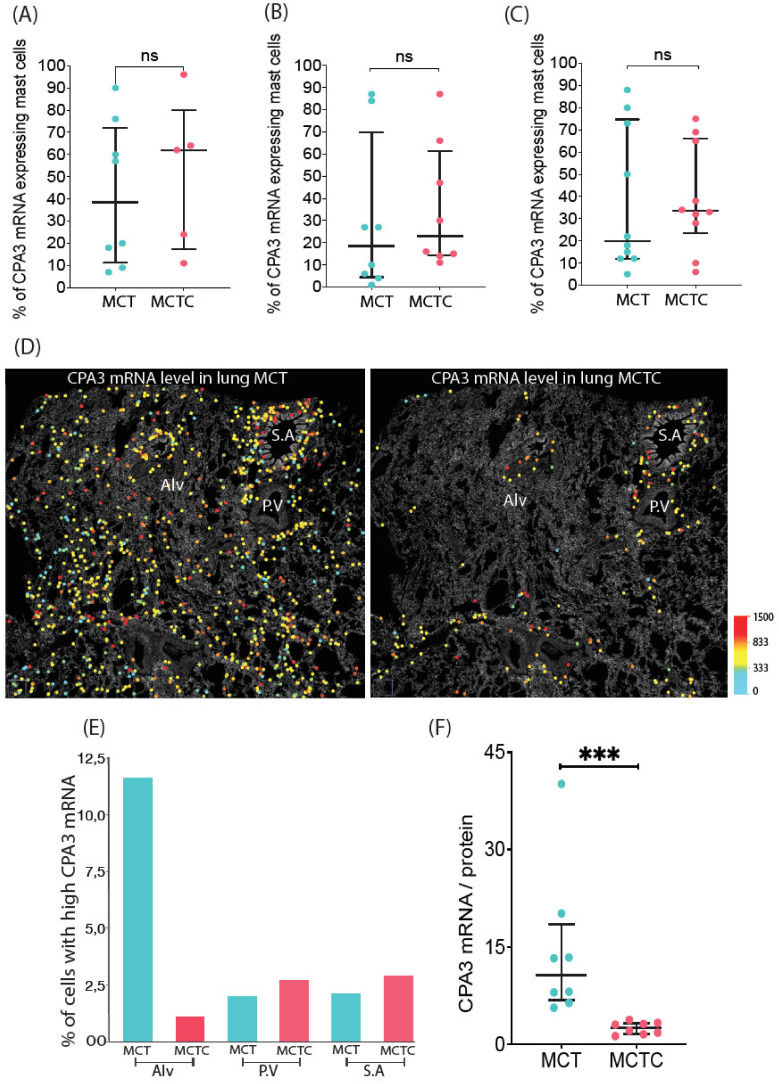
Quantification of CPA3 mRNA expressing mast cell subpopulations within lung anatomical regions. (**A**–**C**) Percentage of mRNA-positive MCT and MCTC populations in (**A**) small airways, (**B**) pulmonary vessels, and (**C**) the alveolar parenchyma. (**D**) Low-magnification spatial distribution maps of CPA3 mRNA expressing MCT and MCTC lung mast cells. For visualization purposes each mast cell has been replaced with enlarged circles that are color coded according to cell mRNA intensity level. (**E**) Comparisons of mean percentage of MCT and MCTC with very high (mean cell intensity > 1200 a.u.) CPA3 mRNA expression in the alveolar region (Alv), pulmonary vessels (P.V) and small airways (S.A). (**F**) The quotient of mean subject CPA3 mRNA/CPA3 protein in total distal lung MCT and MCTC populations. *Statistics:* (**A**,**B**,**F**) The Mann–Whitney test was used to calculate the significance of among mast cell subpopulation in relation to CPA3 protein expression, ns-*p* > 0.05, *** *p* ≤ 0.001.

With lungs having the highest total mRNA expression, this organ was selected for an in-depth analysis looking at sub-organ tissue compartments. This analysis showed that bronchioles (small airways), large pulmonary vessels, and the alveolar parenchyma, all had equal percentages of CPA3-positive cells between their respective MCT and MCTC populations (Figure 4A–C). As revealed by spatial distribution mapping, the dominating lung MCT population had a widespread anatomical distribution whereas, in agreement with our previous observations [29,30,31], MCTC populations were primarily located to small airways and pulmonary vessels (Figure 4D).

In analyzing the distribution of mRNA intensity levels for the > 10,000 individual tissue mast cells analyzed in the lung, a surprising observation was that mast cells expressing very high mRNA intensity levels were found within the MCT population in the alveolar parenchyma, the very region with the lowest amount of CPA3 protein (Figure 4E). Furthermore, in the total distal lung the CPA3 mRNA/protein quotient was significantly higher in MCT cells compared to the MCTC population (Figure 4F).

**Figure 5:**
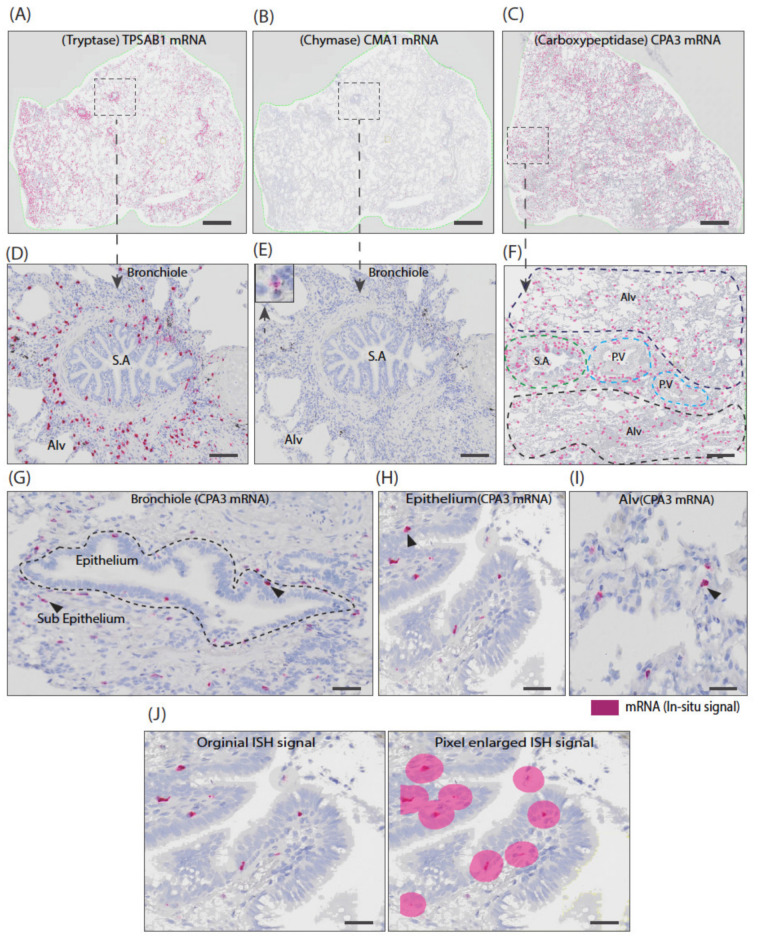
Overview graphic comparisons between tryptase, chymase and CPA3 mRNA expression level and their general spatial distribution in non-diseased lung anatomical regions. (**A**–**C**). Low-magnification overview micrographs of chromogenic in situ hybridization (CISH)-stained lung sections for tryptase (TPSAB1), chymase (CMA1) and carboxypeptidase (CPA3) mRNA. Note that, to allow low-magnification visualization of cells with 10 µm diameters within large cm^2^-size sections, all three markers of ISH signals were enlarged uniformly (as illustrated in **J**). CPA3 mRNA level was similar to the tryptase (TPSAB1) mRNA, whereas chymase (CMA1) mRNA was scarcely found. (**D**–**F**). Zoomed-in areas further illustrate the striking difference in mRNA levels between chymase (CMA1) and CPA3, despite the fact that these are strongly linked at a granule protein level. (**G**–**I**) Examples of original CPA3 ISH signals in bronchiolar and alveolar lung compartments. It is very evident that CPA3 mRNA-expressing mast cells are not restricted to certain lung anatomical regions. (**J**) Images illustrating the original ISH signals being enlarged (using Visiopharm software) used in (**A**–**F**). *Scale bars*; (**A**–**C**) = 200 µm; (**D**–**E**) = 300 µm; 40 µm.

The discrepancy between the close association between CPA3 and chymase at a protein level and the disparate CPA3 and chymase pattern at the mRNA level was also clearly seen in bright field overview in situ hybridization micrographs that, again, revealed an abundance of CPA3 mRNA-positive cells and a pattern that was more like the extensive tryptase mRNA expression than the surprisingly scarce chymase mRNA expression (Figure 5).

### 3.3. Mean Tissue Expression Analysis across Multiple Organs Confirms the Discrepancy between Chymase and CPA3 mRNA Levels in Normal Healthy Lungs

**Figure 6:**
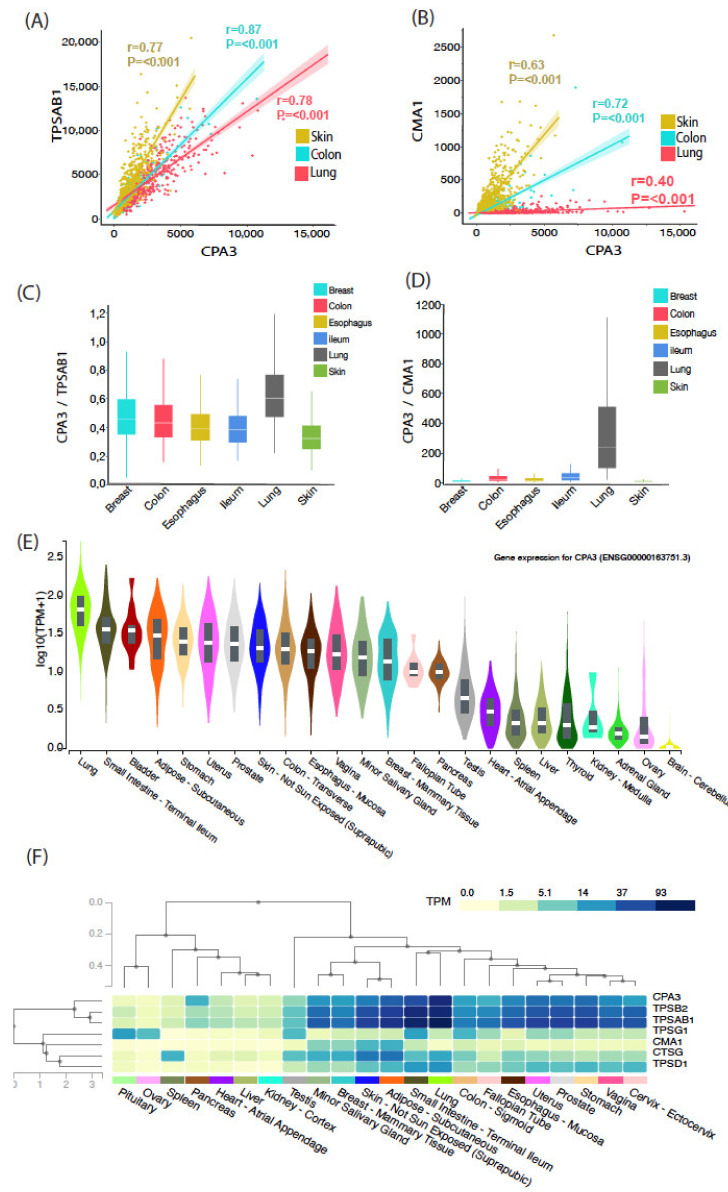
Analysis of tryptase, chymase and CPA3 mRNA expression profile from normalized Gtex gene expression data Tryptase, chymase and CPA3 r-seq data were obtained from Gtex Portal. (**A**,**B**) Mean subject CPA3 mRNA expression in healthy skin, colon and lung was correlated with tryptase (TPSAB1) and chymase (CMA1). (**C**,**D**). Quotients of mean CPA3/TPSAB1 mRNA and CPA3/CMA1 mRNA expression in samples from multiple organs. (**E**). Violin plots showing the CPA3 expression level in 22 human organs (as revealed by Gtex gene query algorithm) (**F**). Relationship between the CPA3 mRNA level and selected mast cell protease genes. Gtex Portal-expression values are shown in Transcripts Per Million (TMP), calculated from a gene model with isoforms collapsed to a single gene.

The present high-end histology-based single-cell analysis of CPA3 is, for practical reasons, restricted to a limited number of study subjects. In order to perform a crude assessment of the correlations between CPA3, tryptase and chymase mRNA expression in a larger population (*n* > 100), we obtained RNA seq raw data from the GTEx open data source [32,33]. After data filtering, we explored the correlation between mean mRNA content for CPA3, tryptase (gene TPSAB1) and chymase (gene CMA1) in non-diseased lung, gut (colon) and skin from hundreds of subjects. The results confirmed that, in the lung, CPA3 mRNA correlates more significantly with tryptase expression than with chymase (Figure 6A,B). Confirming the present novel histological findings, not only was the lung the organ with the highest CPA3 mRNA level, it was also the organ with the highest quotients of CPA3/tryptase (TPSAB1) and CPA3/chymase (CMA1) (Figure 6C–E). Further support for a more general correlation between tryptase expression and CPA3 was confirmed by an extended analysis comparing several MC-related proteases across multiple non-diseased human tissues (Figure 4F).

## 4. Discussion

Compared to tryptase and chymase, the expression pattern of mast cell CPA3 has remained relatively poorly studied. The present detailed histology-based single-cell mapping uncovers a series of novel observations around the nature of CPA3 expression across mast cells in normal tissues. Among these, the markedly disparate patterns of stored CPA3 granule protein and mRNA production in the lung stand out as seemingly surprising. This finding may be biologically relevant and is less unexpected in the light of recent observations of non-typical IgE-independent degranulation and by accepting the fact that mast cell granules are “merely” protein storage sites, where the protease content may not strictly represent which proteases are actually produced or released.

The present study corroborated the expected strong link between granule-stored chymase and CPA3 protein. This tight relationship may be functionally relevant during traditional MC degranulation since chymase endopeptidolytic cleavage of protein/peptide targes may generate a C-terminal aromatic residue cleavable by CPA3 exopeptidase. In any case, it seems that the co-localization of chymase and CPA3 is dictated by shared storage mechanisms as well as a more direct co-dependence for protein granule packaging, as indicated by observations in mice where deletion of the Cpa3 gene resulted in loss of the rodent CMA1 orthologue mMCP-5/cma1 granule protein [29]. Conversely, deletion of mMCP-5/cma1 may lead to loss of mCPA3 [30]. Hence, our present observation of a low-level-stored CPA3 protein in the lung may be a result of absent or low chymase content in the dominating MCT population. From the co-dependence of CPA3 and chymase granule packing, it could be speculated that the high basal expression of CPA3 mRNA revealed here may facilitate the shift from an MCT- to a MCTC-dominating picture which has been observed in distal lungs subjected to chronic inflammation or fibrosis.

Although CPA3 has traditionally been linked to MCTC mast cells, important previous studies have suggested that this association is not entirely strict in the lung. For example, observations in patients with steroid-naïve asthma demonstrated elevated and robust levels of tryptase and CPA3 mRNA in airway epithelial brushings, whereas chymase transcripts were low [23]. Furthermore, a pivotal study in mice comparing mast cell protease profiles across anatomical sites showed that in the skin and intestines, only CTMCs (corresponding to human MCTC) expressed CPA3, whereas in the trachea and bronchi, CPA3 was present in both CTMC and the mucosal mast cell (corresponding to human MCT) [31]. Since mice lack distal lung mast cells, and epithelial brushings only assess intraepithelial mast cells indirectly, the present study represents the first ever detailed and spatially resolved CPA3 mRNA and protein mapping across the entire distal lung MCT and MCTC populations.

In our study, the histological analyses were complemented with investigations into broader mast cell protease gene expression profiles across 22 explored organs. The lung stood out by having the highest number of CPA3 transcripts per million (TPM). Thus, the focus on lung in terms of CPA3 biology is well motivated. As in other gene profiling studies, our GTEx data analysis did not allow for normalization of CPA3 against variations in tissue MC densities among organs and individual study subjects. To mitigate these variations, CPA3 levels were instead normalized against the expression of other MC-specific protease genes in the same samples. The results from this showed that lung mast cells are characterized by a high CPA3 expression relative to other MC proteases and that the CPA3 gene expression is, in contrast to the situation at the protein level, more associated with mRNA expression of tryptase rather than chymase.

This study did not only reveal mast cell differences in CPA3 expression between human organs, but also between anatomical regions within the lung. As shown in both animal models and humans, under healthy baseline conditions, the anatomical compartments of the lung nourish site-specific mast cell phenotypes [31,32,33,34]. In humans, the alveolar mast cells represent the largest but also the least-studied MC pool. Intriguingly, in contrast to the picture in virtually all other tissues, alveolar mast cells lack IgER1 receptors [32,35,36]. Alveolar mast cells are also foremost MCT cells, and are thus thought to lack MRGPRX2, a key receptor for non-IgE mediated degranulation. Consequently, alveolar mast cells may not readily undergo classical degranulation and are likely to be regulated differently than MCs in other anatomical sites. This could be highly rational since the classical physiological responses to mast cell degranulation, like plasma extravasation, would, in the alveolar region, cause lung edema and respiratory failure. Hence, the biological significance of the herein described, finding a high constitutive CPA3 mRNA expression in the distal lung MCT population, will be an important issue for future research.

Our protein analysis showed that the distal lung MCT population virtually lacked CPA3 protein. It is therefore striking that this population not only displayed a robust CPA3 mRNA expression, but that the magnitude of expression intensity was significantly higher than for the corresponding CPA3 protein-laden MCTC population. In general, high mRNA gene expression is coupled to translation and subsequent protein synthesis. A high CPA3 mRNA expression in MCs lacking granule CPA3 may be expected in cells during the refilling phase after an anaphylactic degranulation. However, this is, of course, highly unlikely to explain the results in this study since we explored non-inflamed, albeit former smoke-exposed, directly fixated tissues. Instead, from our data, it seems more reasonable to hypothesize that distal lung MCTs, and likely also MCTCs, already at healthy baseline conditions, produce and release CPA3 without an apparent prior granule storage. Currently, direct evidence for such a non-classical direct protease release is lacking. However, several tentative non-IgE-mediated secretory pathways have been described [37,38]. Alternatively, the high baseline mRNA among MCT cells may not be immediately translated into protein but render the cells capable for a more rapid protein synthesis on demand. In any case, there is now a strong rationale for further studies into the mechanisms controlling CPA3 synthesis and release in the healthy distal lung. As CPA3 targets include a series of potent circulation and matrix-regulating factors [14,15,16,17,18,19], any constitutive or induced CPA3 release from the large MCT population in the lung is likely to have important homeostatic and protective functions.

There are many ways by which a baseline production of CPA3 could have an impact on the lung and the systemic circulation. One such mechanism could be related to the complex relationship between endothelin-1 and mast cells [39], where mast cells have been shown to have important protecting roles by limiting a toxic effect of endothelin-1 [40], possibly through CPA3-mediated endothelin-1 inactivation. As further support for a protective role, decreased serum levels of CPA3 are associated with risk factors of blood vessel disease and cardiovascular damage [41]. Although more research is needed, the proteolytical cleavage of endothelin-1 and other known CPA3 targets, such as neurotensin, angiotensin-1 and apolipoprotein B, further implies potentially broad roles of CPA3 in vascular homeostasis. Naturally, despite its potential role in protection and homeostasis, CPA3 mRNA is upregulated in many diseases [11,13,26,42,43] and may also have more pro-inflammatory or pathogenic actions.

The present study material for our in-depth histology-based analysis was selected to investigate tissues with different inherent MCT/MCTC ratios. The completely reversed balance between CPA3 mRNA-to-protein ratio in the MCT-rich lung and MCTC-dominating skin is a major finding that raises many questions. For example, is the baseline situation in the skin, where low mRNA levels were accompanied by a very high CPA3 granule protein content, a sign of low baseline CPA3 turnover but high capacity to rapidly release high CPA3 on demand? Conversely, and as discussed above, does the high mRNA content in the protein-lacking lung MCT population indicate a high basal CPA3 turnover and continuous baseline CPA3 release? Admittedly, these questions are speculative but nonetheless important, and merit investigation in future separate studies.

The methodological foundation in the present paper is our novel histology-based approach using combined ROI masks to measure the CPA3 content at a single-cell level directly in routine formalin fixated paraffin-embedded (FFPE) samples. The high MC numbers in typical sections thus provide a robust statistical evaluation for this type of analysis. Naturally, for our approach to be valid, the combined tryptase–chymase ROI masks used for CPA3 analysis must be mast-cell-specific. Theoretically, basophils could have affected the measurements, since they are known to express tryptase [44,45]. However, two factors suggest that the influence of basophils was minimal. Firstly, from our previous studies of lung mast cells and basophils [32,46,47], it can be concluded that the basophil numbers in the present type of non-diseased lung tissue are typically less than 1% of the mast cell numbers. Moreover, although basophils may express tryptase, the level of expression is significantly lower than for mast cell tryptase [44,45]. Hence, they will not be picked up by the staining intensity threshold used in the current study for the computerized tryptase segmentation. In contrast to other single-cell staining intensity methods, like flow cytometry, the present histology approach analyses 2D cell profiles rather than whole cells. Accordingly, variations in granule and nucleus areas among cell profiles may contribute to increased fluctuations in the intensity levels. Even so, from a statistical perspective, considering the sheer numbers of analyzed cells, it is clear that this phenomenon has not contributed to our observed CPA3 differences among MC populations. Another technical perspective worth mentioning is the potential bias of differences in tissue processing. To minimize any such technical differences, all the analyzed tissues were processed at our research laboratory, where this done in a consistent and similar fashion independent of tissue source, rather than at the clinical pathology unit, where differences in fixation time, etc., may vary considerably due to logistical issues. Furthermore, for comparisons between the lung MC populations, these were performed simultaneously within the same tissue section, resulting in identical processing conditions. For practical reasons, the patient number was limited. In spite of this, given the clear differences and statistical outcomes, it can be argued that the study material is well justified to allow for robust conclusions around the major observations.

In summary, the present study reveals important new data on baseline mast cell CPA3 dynamics and surprisingly disparate patterns when comparing levels of stored granule protein and mRNA expression. In relation to this, our observations of lung MCT cells with no apparent granule CPA3 protein despite very high CPA3 mRNA levels may reflect a hitherto underestimated baseline of CPA3 production by mucosal MCT cells. Future studies investigating the complex dynamics of CPA3 in health and disease seem highly warranted.

## Data Availability

The data presented in this study are available on request from the corresponding author. For the complementary multi organ gene expression analysis in this study publicly available datasets were analyzed. This raw data can be found here: https://www.gtexportal.org/home.
